# Climatic and Edaphic Drivers of Soil Organic Carbon and Pyrogenic Carbon Stocks Across Elevation and Disturbance Gradients in Colombian Andean Forests

**DOI:** 10.1111/gcb.70135

**Published:** 2025-07-25

**Authors:** Carmen R. Montes‐Pulido, Michael I. Bird, Lidiany C. da Silva Carvalho, Julieth Serrano, Carlos A. Quesada, Ted R. Feldpausch

**Affiliations:** ^1^ School of Agricultural, Livestock, and Environmental Sciences Universidad Nacional Abierta y a Distancia (UNAD) Bogota Colombia; ^2^ College of Science and Engineering and ARC Centre of Excellence for Indigenous and Environmental Histories and Futures James Cook University Smithfield Queensland Australia; ^3^ Geography, Faculty of Environment, Science and Economy University of Exeter Exeter UK; ^4^ Fauna & Flora International Cambridge UK; ^5^ Instituto Nacional de Pesquisas da Amazônia (INPA) Manaus Brazil

**Keywords:** agrosilvopastoral systems, anthropogenic disturbance, elevation gradients, mean annual temperature, pyrogenic carbon, soil, tropical Andes, tropical ecosystem, wildfire

## Abstract

Understanding the drivers of soil organic carbon (SOC) and soil pyrogenic carbon (PyC) variation and their role in natural and managed ecosystems is increasingly important. However, PyC stocks in tropical Andean soils remain understudied. Here, we examined how edaphic and environmental factors affect PyC across elevation and disturbance gradients in 36 plots spanning natural forests and agrosilvopastoral systems in the Colombian Andes. Across the 0–100 cm soil profile, the mean SOC stock in the study region was 433.10 Mg C ha^−1^ (range: 67.97–1462 Mg C ha^−1^), while the mean PyC stock was 34.13 Mg C ha^−1^ (range: 2.29–305.70 Mg C ha^−1^), accounting for approximately ~8% of the total SOC. This PyC stock is approximately nine times greater than the Amazon‐wide average. PyC (%) did not vary significantly with disturbance gradients or soil depths. However, both PyC (%) and SOC (%) varied significantly with elevation zonation (*p* < 0.001). The High Andes had the highest concentrations of PyC (1.3%) and SOC (14.6%), which were substantially higher than the Medium Andes (PyC = 0.17%; SOC = 6.7%) and Low Andes (PyC = 0.06%; SOC = 1.3%). Soil clay content and annual precipitation were the primary drivers of PyC, explaining 56% of the variability when combined with pH, Ca, and NDVI. PyC was positively associated with clay content (Estimate: 0.27, *p* < 0.001) and negatively associated with annual precipitation (Estimate: −0.18, *p* < 0.05). These factors may influence the physical and chemical processes that affect PyC formation and preservation in soils. This analysis provides insight into SOC and PyC variability in Andean forest soils, highlighting the substantial contribution of soil PyC to total soil carbon and its importance as persistent soil carbon under current and predicted warming conditions across the region.

## Graphical abstract

This study examined soil organic carbon (SOC) and pyrogenic carbon (PyC) stocks in tropical Andean soils across elevation and disturbance gradients in Colombia. SOC stocks to 100 cm depth averaged 433.10 Mg C ha^−1^ while PyC was 34.13 Mg C ha^−1^. Key drivers of PyC stocks include soil clay (%), mean annual precipitation (*P*_A_), pH, Ca, and NDVI, which influence PyC formation and preservation. The findings show PyC's significant role in total soil carbon storage and its persistence under climate change, highlighting its importance in natural and managed ecosystems in the Andes.

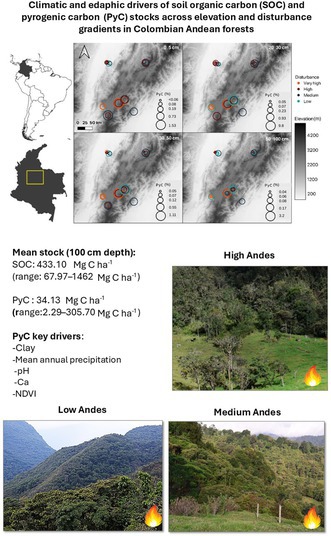

## RESUMEN

Comprender los factores que impulsan la variación del carbono orgánico del suelo (COS) y del carbono pirogénico del suelo (PyC) y su papel en los ecosistemas naturales y gestionados, es cada vez mas importante. Sin embargo, las reservas de PyC en los suelos andinos tropicales siguen estando poco estudiadas. Este estudio evaluó cómo los factores edáficos y ambientales afectan el PyC a través de diferentes gradientes de elevación y perturbación en 36 parcelas que abarcan bosques naturales y sistemas agrosilvopastoriles en los Andes colombianos. En el perfil de suelo de 0–100 cm, el stock medio de COS en la region estudiada fue de 433.10 Mg C ha^−1^ (rango:67.97–1462 Mg C ha^−1^) mientras que la existencia media de PyC fue de 34.13 Mg C ha^−1^ (rango: 2.29–305.70 Mg C ha^−1^) lo que representa aproximadamente el ~8% del COS total. Esta reserva de PyC es aproximadamente nueve veces mayor que la media de toda la Amazonía. El PyC (%) no varió significativamente con los gradientes de perturbación o la profundidad del suelo. Sin embargo, tanto el PyC (%) como el COS (%) variaron significativamente con la zonificación altitudinal (p < 0.001). Los Altos Andes presentaron las concentraciones más altas de PyC (1.3%) y COS(14.6%), respecto a los Andes Medios (PyC = 0.17%; COS = 6.7%) y los Andes Bajos (PyC = 0.06%; COS = 1.3%). El contenido de arcilla del suelo y la precipitación anual fueron los principales factores determinantes del PyC, explicando el 56% de la variabilidad cuando se combinaron con el pH, el Ca y el NDVI. El PyC se asoció positivamente con el contenido de arcilla (estimación: 0.27, *p* < 0.001) y negativamente con la precipitación anual (estimación: −0.18, *p* < 0.05). Estos factores pueden influir en los procesos fisicos y quimicos que afectan a la formacion y conservacion del PyC en los suelos. Este análisis proporciona información sobre la variabilidad del COS y el PyC en los suelos forestales andinos, destacando la sustancial contribucion del PyC al carbono total del suelo y su importancia como carbono persistente en las condiciones de calentamiento actuales y previstas en toda la region.

## 1 Introduction

Soil organic carbon (SOC) forms through the gradual transformation of plant biomass and comprises the largest terrestrial carbon pool, storing three times more carbon than the atmosphere and twice as much as global vegetation (Mayer et al. 2020). Enhancing carbon storage in forests is acknowledged to have the potential to mitigate climate change impacts (Duarte‐Guardia et al. 2020). SOC levels are influenced by vegetation type, climate, and land use history (Jobbágy and Jackson 2000; Berhongaray and Alvarez 2019). Tropical forests account for about one‐third of global SOC stocks (Jackson et al. 2017). However, these stocks show substantial regional variation. Forest soils of the Southwest Amazon have SOC stocks ranging from 16.5 MgC ha^−1^ (Comodoro, Mato Grosso, Brazil) to 96.6 Mg C ha^−1^ (Planaltina, Goias, Brazil) within the 0–30 cm depth interval (Jantalia et al. 2007). In contrast, Andean forests over the same 0–30 cm interval have substantially higher SOC stocks, ranging from 123 to 136 Mg C ha^−1^ (Rolando et al. 2017).

Climate change affects SOC stocks. Tropical land surfaces are predicted to warm by 3°C to 5°C this century (IPCC 2021), with increasing temperature variability and more frequent extreme events (Vogel et al. 2017). Rising temperatures can lead to changes in biotic conditions, including microbial community composition and changes in the availability of substrates for decomposers (Jackson et al. 2017). A study conducted in Barro Colorado (Panama) found that tropical soil emissions are highly sensitive to temperature increase. With two years of experimental warming over soils with a depth range of 0–120 cm, a temperature increase of 4°C increased soil CO_2_ emissions by 55% compared to soils at ambient temperature (Nottingham et al. 2020). In forests of the Colombian Andes, ongoing land use change and increasingly extreme climatic events lead to uncertainty for future SOC dynamics. In high‐altitude Andean ecosystems, land conversion from natural vegetation to crops and grazing, and climate change can accelerate SOC mineralization and loss (Alavi‐Murillo et al. 2022).

A component of the persistent fraction of SOC is soil pyrogenic carbon (PyC), produced from the incomplete combustion and pyrolysis of biomass during fires (Bird et al. 2015). After formation, it is largely resistant to mineralization and will persist for centuries to millennia (Bird et al. 2015). Due to its resistance to degradation (Schmidt et al. 2011), PyC has great potential for long‐term carbon sequestration (Yang et al. 2018). Despite the clear relevance of PyC in the global C cycle, the mechanisms controlling PyC persistence in soils are poorly understood (Santín et al. 2016), and PyC remains an overlooked slow‐cycling carbon stock in global C models (Jones et al. 2019; Volkova et al. 2021). Therefore, including soil PyC in these models could improve the accuracy of global carbon budget estimates (Santín et al. 2016).

A global study found PyC represents an average of 13.7% of the SOC and can be as high as 60% (Reisser et al. 2016). The same study also revealed a significant correlation between climatic conditions—represented by climate zones, mean annual temperature, and precipitation—and the PyC content in the soil (Reisser et al. 2016). The highest percentages of PyC within SOC have been observed in the Boreal region (Ponomarenko and Anderson 2001). Soils with a pH > 7 contain at least 50% more PyC than acidic soils. Furthermore, soil PyC content is significantly correlated with average temperature and precipitation (Reisser et al. 2016). Despite regional advances, there is still uncertainty about soil PyC stocks and distribution throughout the soil profile in different biomes (Gao et al. 2024).

Spatial variation in soil PyC stocks is potentially driven by several variables. PyC stocks may depend on climatic factors (Jauss et al. 2015), vegetation (Lehmann et al. 2008), land use practices (Schmidt et al. 2011), and fires (Bird et al. 2015). PyC accumulation in soil across landscapes may depend on the chemical and physical characteristics of the soil, such as pH and clay content (Reisser et al. 2016; Abney et al. 2019; Cotrufo et al. 2016). PyC resistance to degradation depends on the combustion temperature and time of exposure to a given temperature (Abney et al. 2019). As a degradation‐resistant product, variation in soil PyC also depends on historical fire regimes. In Andean forests, fire is unlikely to occur in the absence of humans due to wet conditions (Bush et al. 2022), with long‐term palaeoecological records of sedimentary charcoal indicating an absence of fire in undisturbed humid Andean forests (Schiferl et al. 2018). Humans have been in the Andes since ~15,500 cal BP (Prates et al. 2020). Their first impacts on ecosystems were, among others, increased fire activity (Bush et al. 2022).

In the Amazon Basin, studies have demonstrated spatial and soil depth‐related variation in PyC (da Silva Carvalho et al. 2018; Koele et al. 2017; de Oliveira et al. 2022), linking this variation with climatic events, human occupation (Oliveira et al. 2020), and past fire, even in areas with no dry season (Feldpausch et al. 2022). *Terra firme* forests in the Amazon store on average 3.62 Mg ha^−1^ PyC over a soil depth of 0–100 cm (Koele et al. 2017). For tropical Andean ecosystems, however, there is little information about the environmental and human drivers of PyC stocks and distribution (Pressler et al. 2022).

The Colombian Andes mountains (*ca* 6°N–23°S) have the highest floral biodiversity in the world. Andean forests contain many unique ecosystems, from tropical rainforest to alpine habitats (Pérez‐Escobar et al. 2022). In an elevation range between 500 and 5400 m, the zonation of Andean Forest structure is mainly defined by elevation, with lower temperature and precipitation at higher altitudes and higher temperature and precipitation at lower altitudes (Rodríguez Eraso et al. 2013). Microclimatic conditions and vegetation are very variable. Almost 38% of the natural ecosystems of the Colombian Andes are intact (Rodríguez Eraso et al. 2013). The soils of the high and middle Andes have Inceptisols and Andisols, while in the low Andes there are Inceptisols and Oxisols. Entisols are occasionally found in the middle and low Andes (IGAC 2001, 2002, 2005). Inceptisols and Andisols are the predominant soil types. Andisols are derived from volcanic ash. Inceptisols show minimal horizon development, while Entisols lack distinct pedogenic horizons (Buol et al. 2011). Oxisols are highly weathered soils with low‐activity clays and iron‐aluminum oxides (Marcelino et al. 2018). This edaphic and climatic variation may be important drivers of differences in SOC and PyC across Andean ecosystems.

To our knowledge, there has been no assessment of PyC stocks in the forest soils of the Colombian Andes, nor are the environmental factors that control PyC distribution known. Consequently, our current understanding of SOC dynamics across the whole Colombian Andes remains incomplete. We hypothesize that soil PyC stocks will be higher in Andean forests and will vary significantly with elevation, as altitude is an indirect indicator of temperature and precipitation (Zhao et al. 2021). Here we analyze PyC stocks in forest soils of the Colombian Andes to address the following questions: (1) How do PyC stocks vary with soil depth, forest disturbance, and elevation? (2) What are the main climatic and environmental factors driving PyC stocks?

## 2 Materials and Methods

### 2.1 Study Site

**FIGURE 1:**
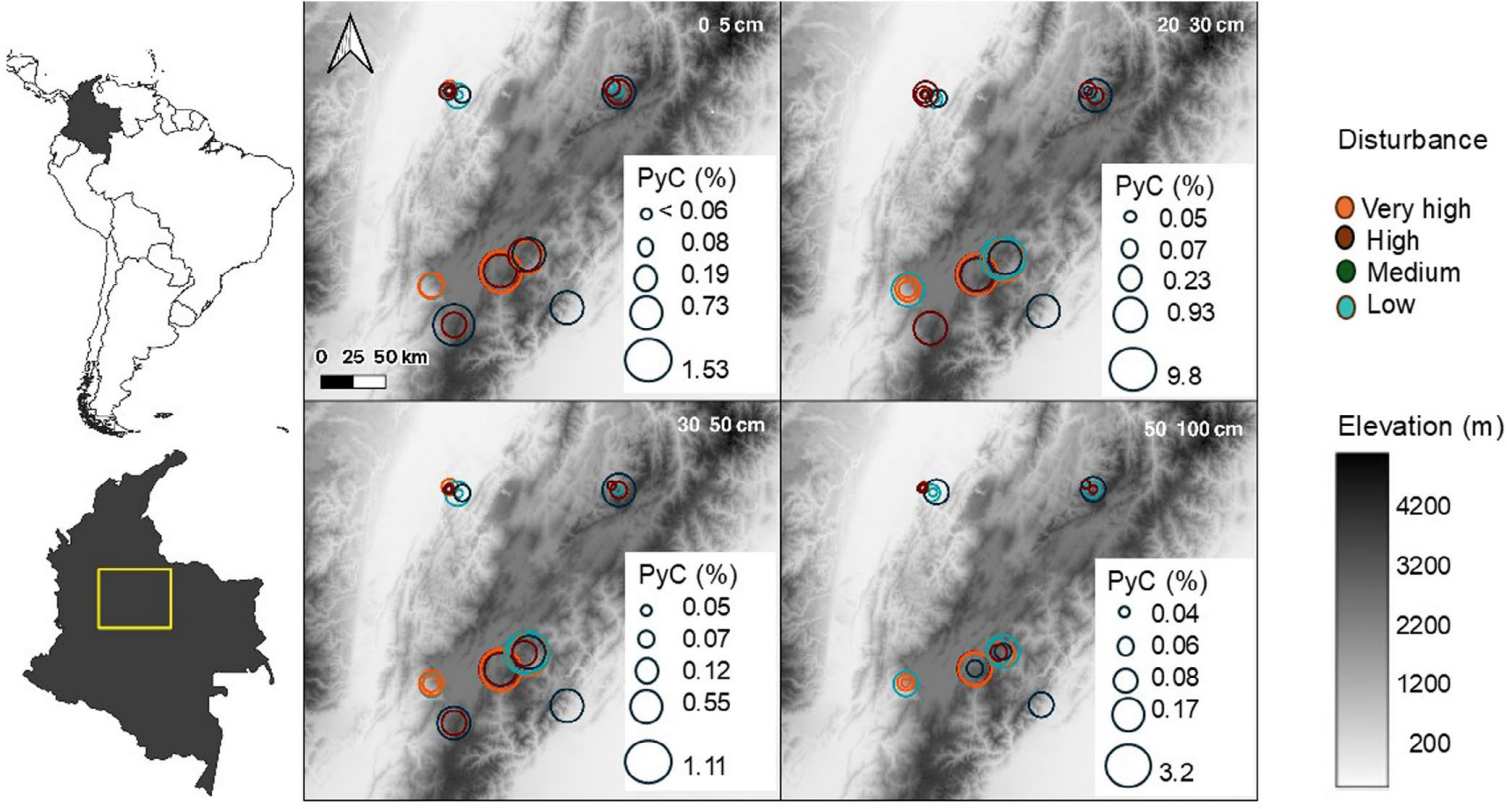
Spatial distribution of PyC (%) soil samples at 0–5, 20–30, 30–50, and 50–100 cm in 36 plots of 0.5‐ha Colombian Andes forest and agrosilvopastoral systems. Circles are scaled to the amount of PyC in percentage and grey shades indicate the altitudinal gradient. Disturbance gradient in the study area: Low (blue), medium (dark green), high (brown), very high (orange). The very high disturbance corresponds to agrosilvopastoral plots. Map lines delineate study areas and do not necessarily depict accepted national boundaries.

The study was conducted in natural forests, in the Colombian Andes, hereafter Andes, at three elevations: high Andes (2736–3139 m), middle Andes (1884–2258 m) and low Andes (176–552 m) (Figure 1), within five nature reserves, Vista Hermosa Regional Nature Reserve, Guatavita, Cundinamarca (4°90′N, 73′W); El Encenillo Nature Reserve, Guasca, Cundinamarca (4°78′N, 73°90′W); Tenasuca Reserve, Tena Cundinamarca (4°68′N, 74°38′W); Cachalú Natural Reserve, El Encino, Santander (6°07′N, 73°13′W), and El Paujil Nature Reserve, Puerto Boyacá, Boyacá (6°02′N, 74°19′W).

The altitudinal gradient examined encompassed the humid forests and páramos of the Andes eastern mountain range. Disturbance gradients in this study are defined as follows: low disturbance areas dominated by old‐growth forests, where trees were selectively logged, and fire was used to remove herbaceous vegetation; medium disturbance sites occupied by agricultural and livestock activities 10–15 years ago, while the high disturbance areas were cleared for farming 16–20 years ago. Within the three disturbance gradients, the sites were abandoned and then allowed to regenerate naturally. Finally, areas with very high disturbance are currently used as agro‐silvopastoral systems. In medium, high, and very high disturbance sites, repeated burning was used to reduce the shrub and herbaceous vegetation cover. Disturbance intensity classification was further refined based on interviews with residents and using satellite images to assess site NDVI variation. Precipitation and temperature vary with elevation (Table 1).

**TABLE 1:** Climate, soil and vegetation characteristics for the three Colombian Andes elevation categories.

Elevation	Altitude	*T*_A_ (°C)	*P*_A_ (mm/year)	Silt (%)	Clay (%)	Sand (%)	Mg (mg/g)	Ca (mg/g)	pH	Soil type	Vegetation
	(masl)	Mean (SD)	Mean (SD)	Mean (SD)	Mean (SD)	Mean (SD)	Mean (SD)	Mean (SD)	Mean (SD)		Most common genus
High Andes	2736–3139	11.3 (±0.8)	1405 (±80)	66.6 (±5.7)	23.8 (±15.6)	9.7 (±10.6)	2.78 (±1.28)	5.33 (±5.68)	4.38 (±0.22)	Inceptisol Andisol Entisol	*Weinmannia* *Brunellia* *Clusia*
Medium Andes	1884–2258	17.08 (±6.7)	1762 (±518)	46.3 (±19.4)	21.7 (±10.3)	32.0 (±23.6)	4.67 (±3.99)	26.49 (±24.32)	4.26 (±0.51)	Inceptisol Andisol Entisol	*Psychotria* *Miconia* *Solanum* *Anthurium*
Low Andes	176–552	27.2 (±4.2)	2597 (±417)	31.7 (±13.4)	17.2 (±7.9)	51.2 (± 19.1)	9.66 (±3.12)	42.85 (±17.87)	4.9 (±0.36)	Inceptisol Entisol Oxisol	*Piper* *Philodendron* *Inga* *Ficus*

Abbreviations: *P*_A_, mean annual precipitation; *T*_A_, mean annual temperature.

### 2.2 Soil Sampling

Soil samples were collected in 27 natural forest plots and 9 agrosilvopastoral plots, each of 0.5 ha (Figure 1). Samples were taken at four points within each plot: two as cores with a one‐piece Edelman auger and two on the outer edge of the plot, where a pit was dug. Subsamples were taken at depths of 0–5, 20–30, 30–50, and 50–100 cm, and did not include the Oh layer (Montes‐Pulido, Feldpausch, et al. 2023).

In the laboratory, all samples were dried at air temperature and sieved at 2 mm to remove roots, detritus, and small rocks. A single composite sample was formed from the four samples per depth (0–5, 20–30, 30–50, and 50–100 cm) and plots. PyC was determined as stable polycyclic aromatic carbon analyzed by hydrogen pyrolysis (HyPy). The abundance of carbon in the sample after HyPy was determined as a proportion of SOC (PyC/SOC). Detailed laboratory measurement methods for PyC are described in Meredith et al. (2012) and summarized here. Samples were loaded with a molybdenum catalyst (~10% dry weight) by means of an aqueous/methanol solution of ammonium dioxydithiomolybdate (NH_4_)_2_MoO_2_S_2_. The dried and catalyst‐loaded samples were placed in a reactor that is pressurized to 150 bar H_2_ under a sweep gas flow of 5 L min^−1^, heated at 300°C min^−1^ up to 250°C, and then intensified at 8°C min^−1^ to a final hold at T of 550°C for 2 min. It is necessary to consider the mass loss of the loaded catalyst during HyPy (Saiz et al. 2018).

Composite samples representing 0–30 cm were used to determine soil chemical and physical properties. A quarter of each sample was taken and homogenized to make the sample. The laboratory analysis of soil physicochemical properties was based on the methodology detailed in Quesada et al. (2010): The soil pH was determined at a ratio of 1:2.5 in H_2_O. Soil particle size distribution was determined by the Bouyoucos method (Gee and Bauder 1986) and each fraction is presented as a percentage. Soil bulk density (BD), taken on site between 0 and 50 cm, with samples taken every 10 cm, was determined from samples collected inside the pits using standard rings of known volume. Samples were then oven‐dried at 105°C to constant weight, crushed and sieved (2 mm) to remove stones and cooled to air temperature before determining final weight. SOC and PyC (in percentage and Mg C ha^−1^) was calculated based on BD, the carbon concentration, and the depth at which the sample was taken.

Total phosphorus was determined by acid digestion at 360°C using concentrated sulphuric acid followed by H_2_O_2_, as described in Tiessen and Moir (1993). Total concentration of Ca, Mg, K, and Na was determined by the silver thiourea method (Ag‐TU; Pleysier and Juo 1980), and the analysis of the filtered extracts using atomic absorption spectrometry. The phosphorus fractionation technique was applied to obtain a measure of available phosphorus. Deionized water, pre‐activated resin strips, and HCl were added to the sieved soil. The solution was then centrifuged, filtered, and diluted. Inorganic phosphorus was separated into Na‐bicarbonate and Na‐hydroxide fractions by acid precipitation from organic matter. The concentration of phosphorus in the filtered solution was measured with a spectrophotometer. Inorganic fractions were then calculated as the difference between the organic fraction and total phosphorus (Tiessen and Moir 1993; Montes‐Pulido, Bird, et al. 2023).

Annual mean temperature (*T*_A_) and precipitation (*P*_A_) data were extracted from WorldClim (Fick and Hijmans 2017). The elevation data was recorded on site with a Garmin Montana 750i GPS. Slope and NDVI data are available from the U.S. Geological Survey (n.d.).

### 2.3 Statistical Analysis

We used spline interpolation (Malone et al. 2009) when necessary to estimate soil bulk density, PyC, and SOC values for the soil depths of 0–5, 5–20, 20–30, 30–50, and 50–100 cm. To compare variations in PyC and SOC across soil depths, disturbance gradients, and elevation zones, we used the non‐parametric Kruskal–Wallis test. To explore the association of environmental climatic data and PyC (%), we first applied Principal Component Analysis (PCA) using all variables to visualize major axes of variation. Subsequently, we assessed potential collinearity among predictor variables of PyC (%) by calculating pairwise Spearman correlation coefficients. Variables showing strong correlation (|*r*| > 0.70) were considered collinear, and one variable from each collinear pair was excluded from regression modeling (Figure S1). We then applied generalized linear models (GLMs) with a Gaussian link function to estimate the relationship between pyrogenic carbon (PyC %) and selected climatic and environmental predictors. Model selection was based on Akaike's Information Criterion (AIC), with lower AIC values indicating better model fit. All statistical analyses were conducted in R version 4.1.3 (R Core Team 2022).

## 3 Results

### 3.1 Variation in SOC and PyC

**FIGURE 2:**
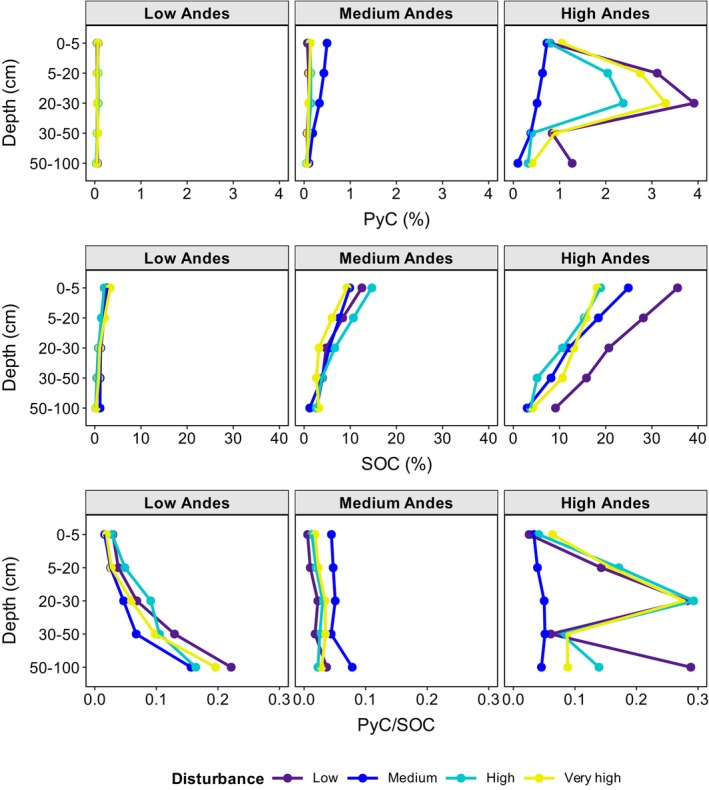
Mean concentration (%) of pyrogenic carbon (PyC) and soil organic carbon (SOC), and PyC/SOC over the sampled interval by depth in 36 forest plots of 0.5 ha in Colombian Andean forest and agrosilvopastoral systems, at three elevations (low: 176–552 m, medium: 1884–2258 m, and high: 2736–3139 m) and four disturbance gradients (purple: Low disturbance; blue: Medium disturbance; green: High disturbance; and yellow: Very high disturbance).

For the entire 0–100 cm soil profile, SOC and PyC stock varied widely across Colombian Andean regions. The overall mean SOC stock was 433.10 Mg C ha^−1^, ranging from 67.97 to 1462 Mg C ha^−1^. PyC represented approximately 8% of the total SOC, with a mean stock of 34.13 Mg C ha^−1^ and a range from 2.29 to 305.70 Mg C ha^−1^ (Table 2 and Figure 2). This substantial variability in total SOC and PyC stocks across the region is primarily attributed to significant changes along the elevation zonation. The highest values of both SOC and PyC were observed in the High Andes, where mean SOC (772.10 Mg C ha^−1^) and PyC (83.27 Mg C ha^−1^) stocks were 6 and 10 times higher, respectively, than in the Low Andes (SOC: 121.28 Mg C ha^−1^; PyC: 8.05 Mg C ha^−1^) (Table 2). The PyC/SOC ratio increased significantly with soil depth (*p* < 0.001), from 0.03 ± 0.02 in the 0–5 cm layer to 0.13 ± 0.15 at 30–50 cm depth (Table 2).

**TABLE 2:** Mean concentration (%) and stocks (Mg ha^−1^) of pyrogenic carbon (PyC), soil organic carbon (SOC) and ratio PyC/SOC in relation to soil depth in the Colombian Andes forests.

Soil depth (cm)	PyC (%)	SOC (%)	PyC stock (Mg/ha)	SOC stock (Mg/ha)	PyC/SOC
0–5	0.37 ± 0.44	12.88 ± 11.42	1.16 ± 1.30	44.20 ± 43.83	0.03 ± 0.02
5–20	0.80 ± 1.69	9.80 ± 9.09	9.80 ± 21.07	116.52 ± 94.98	0.06 ± 0.10
20–30	0.92 ± 2.14	6.71 ± 6.90	8.51 ± 20.25	57.07 ± 51.59	0.11 ± 0.19
30–50	0.26 ± 0.32	4.75 ± 5.14	4.88 ± 5.10	87.45 ± 79.76	0.07 ± 0.04
50–100	0.23 ± 0.56	2.67 ± 3.06	10.67 ± 20.19	139.45 ± 136.74	0.13 ± 0.15

There was no effect of the soil depth (*χ* ^2^ = 8.17, df = 4, *p* = 0.09) or disturbance gradient (*χ* ^2^ = 1.12, df = 3, *p* = 0.77) on the concentration of PyC (%). In contrast, the elevation zonation had a strong influence on PyC (%) (*χ* ^2^ = 111.86, df = 2, *p* < 0.0001). In the High Andes, PyC (1.33% ± 1.32%) was substantially higher compared to the Medium Andes (0.17% ± 0.19%) and Low Andes (0.06% ± 0.01%).

### 3.2 Climatic and Environmental Factors Driving PyC

**FIGURE 3:**
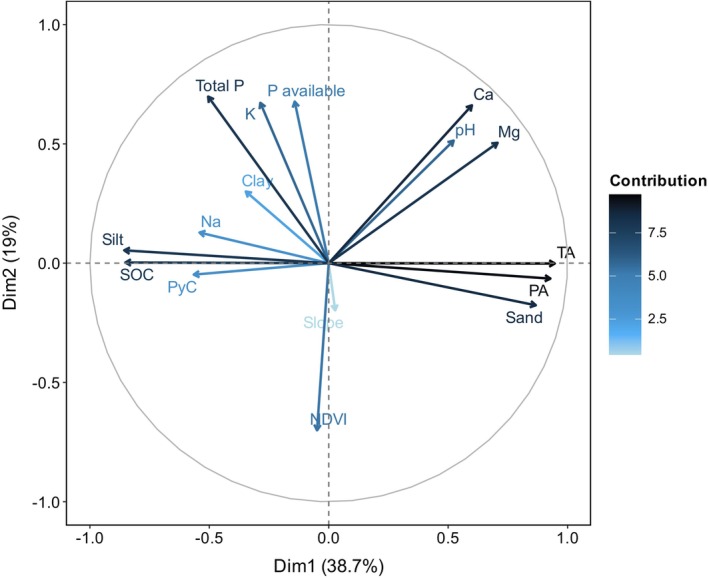
Principal component analysis with soil and climate variables analyzed in the Colombian Andean forests and agrosilvopastoral systems. The highest loadings on the first axis were *T*_A_ (16.9%), *P*_A_ (16.1%), sand (14.0%), and silt (13.2%). The highest loadings on the second axis were Total_p (16.0%), NDVI (15.5%), p_available (14.8%), and Ca^2+^ (13.8%).

We explored patterns among the variables using PCA analysis (Figure 3). The first two dimensions of the PCA explained 58% of the total variation. The variables that contributed strongly to the first dimension were *T*_A_ and *P*_A_. PyC stock changes in relation to temperature and precipitation variations: ~8.05 ± 2.24 Mg C ha^−1^ at 27°C and 2597 mm; ~11.06 ± 6.92 Mg C ha^−1^ at 17°C and 1762 mm, and ~83.27 ± 86.57 MgC ha^−1^ at 11°C and 1405 mm (Table 3). Climatic drivers of PyC were followed in importance by sand and silt, with only silt being negative. Total P, K, NDVI, available phosphorus, Ca^2+^, and pH had a strong effect on the second dimension with a negative correlation for total phosphorus, K, NDVI, and available phosphorus. Based on a GLM analysis (Table 4), clay content and *P*_A_ were the most important predictors of PyC accumulation. Clay content showed a significant positive relationship with PyC (*β* = 0.27, *p* < 0.01), while *P*_A_ had a significant negative relationship (*β* = −0.18, *p* = 0.03). Additional variables including pH, calcium content, and NDVI contributed to model performance (AIC = 34.98, pseudo‐*R* ^2^ = 0.56) but were not statistically significant predictors.

**TABLE 3:** Mean concentrations (%) and total stocks (Mg ha^−1^) 0–100 cm soil depth of pyrogenic carbon (PyC), soil organic carbon (SOC) and ratio PyC/SOC in relation to elevation zone.

Zones	PyC (%)	SOC (%)	PyC stock (Mg/ha) 0–100 cm	SOC stock (Mg/ha) 0–100 cm	PyC/SOC
Low Andes	0.06 ± 0.01	1.33 ± 0.60	8.05 ± 2.24	121.28 ± 75.23	0.08 ± 0.03
Medium Andes	0.17 ± 0.19	6.65 ± 4.60	11.06 ± 6.92	405.81 ± 191.09	0.03 ± 0.02
High Andes	1.33 ± 1.32	14.57 ± 6.12	83.27 ± 86.57	772.10 ± 292.30	0.12 ± 0.13

**TABLE 4:** Estimated regression parameters, standard errors, 95% confidence intervals, *t*‐value, and *p*‐values for the general linear model function quantifying the relationship between pyrogenic carbon (PyC) concentration (%) and climatic and environmental factors.

	Estimate	SE	*t*	*p*
Intercept	−0.09	0.06	−1.41	0.17
*P*_A_	−0.18	0.08	−2.31	0.03*
pH	0.14	0.08	1.85	0.07
Ca	−0.13	0.09	−1.44	0.16
NDVI	0.10	0.07	1.43	0.16
Clay	0.27	0.07	3.99	0.00**

*Note:* Data from 36 forest and agrosilvopastoral plots of 0.5 ha in the Colombian Andean Forest at three elevations. Values with asterisks (*) are significant: (*) *p*‐value < 0.05; (**) *p*‐value < 0.01.

## 4 Discussion

Research is increasingly expanding our understanding of the drivers of variation in PyC stocks and distribution as well as its role in the carbon cycle of natural and managed ecosystems (Gale and Thomas 2021; Pressler et al. 2022). While soil PyC has been estimated for lowland tropical forests spanning the Amazon Basin (Koele et al. 2017; de Oliveira et al. 2022), there is a lack of data for tropical Andean forests. In one of the first studies of the drivers of soil PyC stocks across elevation and disturbance gradients in Colombian Andean forests, we show that soil PyC distribution is primarily driven by clay content and *P*_A_. Our findings reveal that PyC stocks were significantly higher in the high Andean forests compared to medium and low elevation forests. The results show a substantial contribution of PyC to total SOC stocks and therefore its potential role as a persistent carbon pool under global warming.

### 4.1 Colombian Andean SOC and PyC Stocks

Colombian Andean soils demonstrated great carbon storage capacity, mainly in the High Andes, where mean stocks of SOC (772.10 Mg C ha^−1^) and PyC (83.27 Mg C ha^−1^) significantly exceed values reported for other Neotropical systems. The mean SOC stock to a depth of 100 cm observed in this study (433.10 Mg C ha^−1^) across the low, middle, and high Andes falls within the range (60–600 Mg C ha^−1^) reported in a meta‐analysis of moist tropical forests (Raich et al. 2006) and is consistent with values for Bolivian tropical montane rainforests (220–530 Mg C ha^−1^) (Schawe et al. 2007). The total average PyC for the three elevation zones (8.05–83.27 MgC ha^−1^ to 0–100 cm) was higher than estimates for seasonal forest fragments in Roraima, Brazil (0.46–4.69 Mg C ha^−1^ to 0–100 cm) (Turcios et al. 2016), three forest types in the northern Brazilian Amazon (2.44 Mg C ha^−1^ to 0–100 cm) (da Silva Carvalho et al. 2018) and the general mean for the entire Amazon (3.62 Mg C ha^−1^ to 0–100 cm) (Koele et al. 2017). Our regional average estimate of 34.13 Mg C ha^−1^ indicates that PyC stocks in Colombian Andean forests are approximately nine times greater than the Amazon‐wide average. The PyC/SOC ratio of approximately 8% across the study region indicates that pyrogenic carbon represents a significant fraction of total soil carbon storage. This proportion is notably higher than typically reported for tropical lowland systems (2%–5%) (Koele et al. 2017), suggesting the higher PyC production and accumulation capacity of these fire‐prone montane environments.

### 4.2 SOC and PyC Vertical Variability

The decline in concentrations of soil organic carbon (SOC %) with increasing soil depth, particularly in the high and middle Andean regions (Figure 2), aligns with findings from other Andean and Amazonian ecosystems (Zimmermann et al. 2012; Koele et al. 2017). This vertical distribution is likely driven by the greater abundance of roots in upper soil layers at higher elevations, where herbs and shrubs contribute substantially to belowground biomass, thereby enhancing SOC accumulation near the surface (Jobbágy and Jackson 2000). Additionally, inputs of lignin‐rich aboveground biomass combined with relatively low decomposition rates may further promote SOC retention in surface horizons (Austin and Vitousek 1998). Conversely, SOC and PyC stocks exhibited an increasing trend with depth (Table 2), a pattern potentially driven by the elevated bulk density commonly associated with soils under livestock grazing regimes (Patiño et al. 2021).

The vertical distribution of PyC varied heterogeneously with depth across elevations (Figure 2). The absence of significant differences between depth ranges for PyC (%) may be influenced by physical processes such as anthroperturbation, where soil mixing due to agricultural activities can redistribute particulate matter, including PyC (Hobley 2019). Practices such as deep tillage transfer organic matter and PyC from the surface to the subsoil (Alcántara et al. 2016), leading to a more uniform PyC distribution across depths. However, the greatest PyC concentration was observed in the layer beneath the surface horizon (20–30 cm), especially in the High Andes (Figure 2), likely due to vertical transport within the soil profile (Rumpel et al. 2015). High PyC abundance in the deeper soil layers necessarily indicates translocation from the upper layers, as it was formed above or close to the soil surface. Volcanic soils in the high Andes, characterized by excellent aeration and infiltration, likely promote the movement and redistribution of PyC to the subsurface, facilitated by water flow (Klein et al. 2008; Hilscher and Knicker 2011; Bellè et al. 2021). Similar patterns were observed in the Chilean Andes in soils under forests exposed to severe fire (Rivas et al. 2012) and Australian cattle grazing lands (Qi et al. 2017). PyC translocation is likely affected by a combination of factors, including rainfall infiltration (Abney et al. 2019; Cotrufo et al. 2016), soil texture, biological activity, and land use such as tillage (Dai et al. 2005; Zhan et al. 2013). These findings suggest that, although PyC is primarily generated at the surface, its vertical translocation may facilitate long‐term sequestration in subsoil horizons, where physical stabilization mechanisms provide protection against degradation (Matosziuk et al. 2020).

### 4.3 Main Climatic and Environmental Factors Driving PyC Distribution

The PyC stocks observed at higher elevations were approximately ten times greater than those at lower elevations, driven by distinct edaphic and climatic conditions along the elevation gradient. Our results are consistent with findings from other regions, where lower precipitation levels have been associated with reduced PyC storage compared to areas with higher precipitation (Reisser et al. 2016). High‐elevation sites exhibited higher clay content (24%) and lower annual precipitation (1405 mm) compared to the lowland sites (clay: 17%; precipitation: 2597 mm). The elevated clay content likely contributes to enhanced PyC retention through increased adsorption of organic carbon compounds onto clay surfaces. This process is facilitated by the high specific surface area of clay particles and the presence of polyvalent cations, which promote the formation of stable organo‐mineral complexes. These complexes play a crucial role in protecting PyC from microbial and enzymatic degradation (Czimczik and Masiello 2007). In contrast, PyC oxidation is promoted by alternating wet‐dry conditions (Nguyen and Lehmann 2009). Additionally, slower decomposition rates due to lower precipitation and temperature at high elevations may slow decomposition rates, further contributing to PyC accumulation (Zaffar and Sheng‐Gao 2015; De la Cruz‐Amo et al. 2020; Dieleman et al. 2013). In the middle and lower Andes, environmental conditions (low clay content, high precipitation and temperature) likely favor PyC decomposition and oxidation. Flooding is especially common in the lower elevation sites, particularly in the Magdalena River Valley, where the lowland plots are located. Such hydrological dynamics may enhance PyC leaching and decomposition, thereby reducing its persistence in the soil (Johnson et al. 2007; Berhe 2012; Doetterl et al. 2012).

### 4.4 Land Use, Historical Fire Regimes and Long‐Term PyC Accumulation

The absence of significant relationships between current disturbance gradients and PyC stocks provides important insights into the temporal dynamics of pyrogenic carbon accumulation. PyC derived from plant sources, such as natural grasslands, tree wood, crops, and pastures, retains a significant portion of the original plant cell architecture, resulting in a highly porous material that is susceptible to flotation (Brewer et al. 2014). Under such conditions, processes that enhance the stability of PyC in soil include interactions with soil minerals, microbial activity, and soil water dynamics. Each of these processes operates on distinct temporal scales, with hydrological cycle interactions occurring most rapidly and exhibiting variability across different ecosystems (Masiello and Berhe 2020). PyC movement from surface to mineral soil starts < 1 year after (Matosziuk et al. 2020) and a significant part of the recently produced PyC may be locally redistributed and accumulated at depths of 20–30 cm, particularly at higher elevations. Current PyC distributions, therefore, largely represent a legacy of historical fire regimes rather than recent burning activities (Peter‐Contesse et al. 2024), creating a temporal mismatch between contemporary disturbance patterns and observed soil carbon stocks.

Fire frequency in the northern Andes was low during the Pleistocene (Van der Hammen et al. 1973) but increased with pre‐Columbian human occupation around 15,500 years ago (Prates et al. 2020). Indigenous people used fire for resource management and landscape modification (Gao et al. 2024), mainly through slash‐and‐burn agriculture (Patiño 1965; Jantz and Behling 2012). Fire use intensified in the early 16th century and has further increased since the mid‐20th century (Castilla‐Beltrán et al. 2018; Koch et al. 2019), with fires now occurring every 2 to 10 years due to agricultural expansion (Molano 1996). Between 2001 and 2013, an average of 4399 ha per year was burned in the páramo complex of Cruz Verde‐Sumapaz with moderate fire severity (∆NBR 270–439) accounting for 41% of the affected area (Borrelli et al. 2015).

The exceptionally high PyC stocks in high Andean sites likely represent the cumulative effect of this long‐term burning history, particularly in páramo ecosystems where continuous human influence spans over centuries. These landscapes have experienced repeated burning cycles for agricultural preparation, livestock management, and mining expansion (Etter et al. 2008), creating substantial PyC inputs that have been preserved under favorable climatic and edaphic conditions.

### 4.5 Vegetation‐Fire Interactions Across Elevation Zones

The regional fire history, macroclimate, and environmental conditions characterizing forest types may influence the type and the amount of combustible material available (Armenteras‐Pascual et al. 2011; da Silva et al. 2024) and converted to PyC. Fire susceptibility in the tropical Andes varies along the altitudinal gradient with vegetation composition, fuel load distribution, and humidity (Armenteras et al. 2020), creating distinct PyC formation environments that help explain observed distribution patterns. High humidity can inhibit fire spread, while dry seasons lead to extensive fires. Strong solar radiation further increases fire risk by drying vegetation and litter (Román‐Cuesta et al. 2014).

Contemporary fire activity varies throughout the year, with some studies reporting a high concentration of fires during the first dry season (January–March) (FIRMS 2015), while others observe a bimodal distribution with a secondary peak during the second dry season (July–September) (Amaya Villabona and Armenteras Pascual 2012). In the lower elevations of the high Andes, fire events are particularly common in August (Borrelli et al. 2015).

High Andean páramo ecosystems represent particularly fire‐prone environments due to their climate characteristics, vegetation structure, and composition. Dense stands of *frailejonales* (*Espeletia* sp.) and natural grasslands ( *Calamagrostis effusa* Steud) create high fuel loads with readily combustible materials (Cardoso and Schenetter 1976). The presence of thick organic horizons provides substantial combustible material that can sustain fires in the drought season and high PyC production even without significant woody biomass fuel. Additionally, controlled burning practices in the High Andes are used to promote palatable grass growth for livestock and prepare areas for potato and pea cultivation (Hofstede 1995). These management fires tend to be less intense than wildfire events but occur with greater frequency, creating consistent PyC inputs over extended periods.

In the natural forests of the middle and lower Andes, tree vegetation is more abundant compared to the higher Andes, where human‐induced fires frequently occur during dry periods (Villabona and Pascual 2012). Post‐fire mortality among certain tropical tree species is common (Brando et al. 2019), with rates ranging from 5% to 90% (McDowell et al. 2018). This wide variability is driven by factors influencing fire intensity, such as fuel load, microclimatic conditions, fire season duration, and extreme climate events (Nepstad et al. 2001). In fire‐affected areas, reduced tree growth may lead to lower biomass production (Rappaport et al. 2018), ultimately decreasing PyC formation. At lower elevations, human‐induced fires are likely to generate high soil temperatures. The formation temperature of pyrogenic carbon (PyC) critically influences its erosion potential through temperature‐dependent particle size differentiation. High‐temperature PyC formation typically yields smaller particulate fractions compared to low‐temperature production (Bruun et al. 2011; Kim et al. 2012). Furthermore, PyC mobility is mediated by intrinsic physicochemical properties including composition and porosity, fire‐induced hydrophobicity changes, and the vertical redistribution of mobile organic compounds (MOCs). During combustion, MOCs volatilize from surface soils and subsequently condense on hydrophobic substrates in cooler subsurface layers (20–30 cm depth), potentially facilitating the lateral transport of post‐fire PyC surface residues (Abney and Berhe 2018; Robichaud 2000).

## 5 Limitations and Future Research

Our study, based on 36 plots spanning elevation and disturbance gradients in natural forests and silvopastoral systems, provides strong foundational research on drivers of SOC and PyC in the Colombian Andes. Future analysis could extend to the Orinoco and Amazonian piedmont regions, as well as the dry forests of the intermontane valleys, to validate the observed patterns.

While the disturbance gradient is useful for comparisons, incorporating more precise and quantifiable metrics, such as those related to fire regime, could offer a clearer understanding of how human activities influence PyC and SOC stocks. Additionally, this work does not extensively examine the interactions of PyC with other soil components, such as microbial communities, mineral content, or other forms of organic matter that may influence PyC dynamics. Further analysis should focus on these interactions to gain a more comprehensive understanding. In addition, this study offers a snapshot of PyC and SOC stocks across elevation and disturbance gradients but does not consider temporal variations. SOC levels may fluctuate in response to climate variability, seasonal changes, or long‐term ecosystem dynamics.

Currently, there is no standardized approach for PyC analysis and other approaches may yield different estimates of PyC stocks. Pyrogenic carbon (PyC) was determined here as stable polycyclic aromatic carbon by hydrogen pyrolysis (HyPy). This technique has proven to be very effective in characterizing PyC abundance (Meredith et al. 2012). We are confident that PyC is not overestimated, as the technique quantifies only the most recalcitrant PyC, not all carbon of pyrogenic origin, and the method takes into account the mass loss of the loaded catalyst during the HyPy process. In addition, a production correction was applied during the procedure, where the carbon abundance in the sample after HyPy is corrected for the known, minimal, potential charring of SOC during the process.

## 6 Conclusions

In the Colombian Andean forests, one of the earliest regions in South America to be colonized by humans (by ~15,500 cal BP, Prates et al. 2020), we found that PyC stocks per hectare are ~9 times higher than those of the entire Amazon region. The highest PyC stocks were found in the high Andes. We also observed high PyC stocks in the subsurface horizons, especially in the high Andes, suggesting PyC is mobilized by abiotic and/or biotic factors from the surface to deeper soil layers. Our results indicate strong climatic and edaphic controls on soil PyC, likely also modified by historical land use through pre‐history. Soil conservation and management are important to reduce the risk to PyC and SOC stocks due to climate warming, land‐use change, and erosion from the steep slopes throughout the Andes. Our findings on drivers in relation to altitudinal variation can improve estimates of the long‐term fate of soil PyC as a globally important regional resistant C pool.

## Author Contributions

**Carmen R. Montes‐Pulido:** data curation, formal analysis, investigation, software, visualization, writing – original draft, writing – review and editing. **Michael I. Bird:** resources, writing – review and editing. **Lidiany C. da Silva Carvalho:** software, writing – review and editing, resources. **Julieth Serrano:** project administration, writing – review and editing. **Carlos A. Quesada:** resources. **Ted R. Feldpausch:** conceptualization, funding acquisition, methodology, project administration, supervision, writing – review and editing.

## Conflicts of Interest

The authors declare no conflicts of interest.

## Supporting information


Data S1.

## Data Availability

The data and code that support the findings of this study are openly accessible in the Zenodo repository at https://doi.org/10.5281/zenodo.14926032 and https://doi.org/10.5281/zenodo.14926046. Soil data are available at https://doi.org/10.5285/fbcfc877‐a38e‐4c26‐8d8b‐7226392493db. Slope and Normalized Difference Vegetation Index (NDVI) data were derived from the Landsat 9 Satellite imagery, accessed through the USGS Earth Explorer platform (https://earthexplorer.usgs.gov/, https://doi.org/10.5066/P975CC9B). Climate data were sourced from the WorldClim database, specifically the WorldClim 2.1 dataset (https://www.worldclim.org/data/worldclim21.html).

## References

[gcb70135-bib-0001] Abney, R. B. , and A. A. Berhe . 2018. “Pyrogenic Carbon Erosion: Implications for Stock and Persistence of Pyrogenic Carbon in Soil.” Frontiers in Earth Science 6: 26. 10.3389/feart.2018.00026.

[gcb70135-bib-0002] Abney, R. B. , T. J. Kuhn , A. Chow , W. Hockaday , M. L. Fogel , and A. A. Berhe . 2019. “Pyrogenic Carbon Erosion After the Rim Fire, Yosemite National Park: The Role of Burn Severity and Slope.” Journal of Geophysical Research 124, no. 2: 432–449. 10.1029/2018JG004787.

[gcb70135-bib-0003] Alavi‐Murillo, G. , J. Diels , J. Gilles , and P. Willems . 2022. “Soil Organic Carbon in Andean High‐Mountain Ecosystems: Importance, Challenges, and Opportunities for Carbon Sequestration.” Regional Environmental Change 22, no. 4: 128. 10.1007/s10113-022-01980-6.

[gcb70135-bib-0004] Alcántara, V. , A. Don , R. Well , and R. Nieder . 2016. “Deep Ploughing Increases Agricultural Soil Organic Matter Stocks.” Global Change Biology 22, no. 8: 2939–2956. 10.1111/gcb.13289. 26994321

[gcb70135-bib-0005] Amaya Villabona, D. , and D. Armenteras Pascual . 2012. “Incidencia de incendios sobre la vegetación de Cundinamarca y Bogotá DC (Colombia), entre 2001 y 2010.” Acta Biológica Colombiana 17, no. 1: 143–158.

[gcb70135-bib-0006] Armenteras, D. , T. M. González , O. Vargas Ríos , M. C. Meza Elizalde , and I. Oliveras . 2020. “Incendios en ecosistemas del norte de Suramérica: avances en la ecología del fuego tropical en Colombia, Ecuador y Perú.” Caldasia 42, no. 1: 1–16. 10.15446/caldasia.v42n1.77353.

[gcb70135-bib-0007] Armenteras‐Pascual, D. , J. Retana‐Alumbreros , R. Molowny‐Horas , R. M. Roman‐Cuesta , F. Gonzalez‐Alonso , and M. Morales‐Rivas . 2011. “Characterising Fire Spatial Pattern Interactions With Climate and Vegetation in Colombia.” Agricultural and Forest Meteorology 151, no. 3: 279–289. 10.1016/j.agrformet.2010.11.002.

[gcb70135-bib-0008] Austin, A. T. , and P. M. Vitousek . 1998. “Nutrient Dynamics on a Rainfall Gradient in Hawaii.” Oecologia 113: 519–529. 10.1007/s004420050405. 28308032

[gcb70135-bib-0009] Bellè, S. L. , A. A. Berhe , F. Hagedorn , et al. 2021. “Key Drivers of Pyrogenic Carbon Redistribution During a Simulated Rainfall Evento.” Biogeosciences 18, no. 3: 1105–1126. 10.5194/bg-18-1105-2021.

[gcb70135-bib-0010] Berhe, A. A. 2012. “Decomposition of Organic Substrates at Eroding vs. Depositional Landform Positions.” Plant and Soil 350, no. 1: 261–280. 10.1007/s11104-011-0902-z.

[gcb70135-bib-0011] Berhongaray, G. , and R. Alvarez . 2019. “Soil Carbon Sequestration of Mollisols and Oxisols Under Grassland and Tree Plantations in South America—A Review.” Geoderma Regional 18: e00226. 10.1016/j.geodrs.2019.e00226.

[gcb70135-bib-0012] Bird, M. I. , J. G. Wynn , G. Saiz , C. M. Wurster , and A. McBeath . 2015. “The Pyrogenic Carbon Cycle.” Annual Review of Earth and Planetary Sciences 43: 273–298. 10.1146/annurev-earth-060614-105038.

[gcb70135-bib-0013] Borrelli, P. , D. Armenteras , P. Panagos , S. Modugno , and B. Schütt . 2015. “The Implications of Fire Management in the Andean Paramo: A Preliminary Assessment Using Satellite Remote Sensing.” Remote Sensing 7, no. 9: 11061–11082. 10.3390/rs70911061.

[gcb70135-bib-0014] Brando, P. M. , D. Silvério , L. Maracahipes‐Santos , et al. 2019. “Prolonged Tropical Forest Degradation due to Compounding Disturbances: Implications for CO_2_ and H_2_O Fluxes.” Global Change Biology 25, no. 9: 2855–2868. 10.1111/gcb.14659. 31237398

[gcb70135-bib-0015] Brewer, C. E. , V. J. Chuang , C. A. Masiello , et al. 2014. “New Approaches to Measuring Biochar Density and Porosity.” Biomass and Bioenergy 66: 176–185. 10.1016/j.biombioe.2014.03.059.

[gcb70135-bib-0016] Bruun, E. W. , H. Hauggaard‐Nielsen , N. Ibrahim , et al. 2011. “Influence of Fast Pyrolysis Temperature on Biochar Labile Fraction and Short‐Term Carbon Loss in a Loamy Soil.” Biomass and Bioenergy 35, no. 3: 1182–1189. 10.1016/j.biombioe.2010.12.008.

[gcb70135-bib-0017] Buol, S. W. , R. J. Southard , R. C. Graham , and P. A. Mc Daniel . 2011. Soil Genesis and Classification. 6th ed, 543. John Wiley and Sons.

[gcb70135-bib-0018] Bush, M. B. , A. Rozas‐Davila , M. Raczka , et al. 2022. “A Palaeoecological Perspective on the Transformation of the Tropical Andes by Early Human Activity.” Philosophical Transactions of the Royal Society, B: Biological Sciences 377, no. 1849: 20200497. 10.1098/rstb.2020.0497. PMC8899620 35249394

[gcb70135-bib-0019] Cardoso, H. , and M. L. Schenetter . 1976. “Estudios ecológicos en el páramo de Cruz Verde, Colombia. III. La biomasa de tres asociaciones vegetales y la productividad de *Calamagrostis effusa* (H.B.K.) Steud y *Paepallanthus columbiensis* Ruhl. en comparación con la concentración de clorofila.” Caldasia 11, no. 54: 69–83.

[gcb70135-bib-0020] Castilla‐Beltrán, A. , H. Hooghiemstra , M. L. Hoogland , et al. 2018. “Columbus' Footprint in Hispaniola: A Paleoenvironmental Record of Indigenous and Colonial Impacts on the Landscape of the Central Cibao Valley, Northern Dominican Republic.” Anthropocene 22: 66–80. 10.1016/j.ancene.2018.05.003.

[gcb70135-bib-0021] Cotrufo, M. F. , C. Boot , S. Abiven , et al. 2016. “Quantification of Pyrogenic Carbon in the Environment: An Integration of Analytical Approaches.” Organic Geochemistry 100: 42–50. 10.1016/j.orggeochem.2016.07.007.

[gcb70135-bib-0022] Czimczik, C. I. , and C. A. Masiello . 2007. “Controls on Black Carbon Storage in Soils.” Global Biogeochemical Cycles 21, no. 3. 10.1029/2006GB002798.

[gcb70135-bib-0023] da Silva Carvalho, L. C. , P. M. Fearnside , M. T. Nascimento , and R. I. Barbosa . 2018. “Amazon Soil Charcoal: Pyrogenic Carbon Stock Depends of Ignition Source Distance and Forest Type in Roraima, Brazil.” Global Change Biology 24, no. 9: 4122–4130. 10.1111/gcb.14277. 29668042

[gcb70135-bib-0024] da Silva, L. J. , L. C. Silva Oliveira , G. N. Nóbrega , et al. 2024. “Pyrogenic Carbon Stocks Are Controlled by Hydro‐Edaphic Conditions in Different Forest Types of the Northern Brazilian Amazon.” Catena 236: 107731. 10.1016/j.catena.2023.107731.

[gcb70135-bib-0025] Dai, X. , T. W. Boutton , B. Glaser , R. J. Ansley , and W. Zech . 2005. “Black Carbon in a Temperate Mixed‐Grass Savanna.” Soil Biology and Biochemistry 37, no. 10: 1879–1881. 10.1016/j.soilbio.2005.02.021.

[gcb70135-bib-0027] De la Cruz‐Amo, L. , G. Bañares‐de‐Dios , V. Cala , et al. 2020. “Trade‐Offs Among Aboveground, Belowground, and Soil Organic Carbon Stocks Along Altitudinal Gradients in Andean Tropical Montane Forests.” Frontiers in Plant Science 11: 106. 10.3389/fpls.2020.00106. 32194581 PMC7062916

[gcb70135-bib-0028] de Oliveira, E. A. , T. R. Feldpausch , B. S. Marimon , et al. 2022. “Soil Pyrogenic Carbon in Southern Amazonia: Interaction Between Soil, Climate, and Above‐Ground Biomass.” Frontiers in Forests and Global Change 5: 880963. 10.3389/ffgc.2022.880963.

[gcb70135-bib-0029] Dieleman, W. I. , M. Venter , A. Ramachandra , A. K. Krockenberger , and M. I. Bird . 2013. “Soil Carbon Stocks Vary Predictably With Altitude in Tropical Forests: Implications for Soil Carbon Storage.” Geoderma 204: 59–67. 10.1016/j.geoderma.2013.04.005.

[gcb70135-bib-0031] Doetterl, S. , J. Six , B. Van Wesemael , and K. Van Oost . 2012. “Carbon Cycling in Eroding Landscapes: Geomorphic Controls on Soil Organic C Pool Composition and C Stabilization.” Global Change Biology 18, no. 7: 2218–2232. 10.1111/j.1365-2486.2012.02680.x.

[gcb70135-bib-0032] Duarte‐Guardia, S. , P. Peri , W. Amelung , et al. 2020. “Biophysical and Socioeconomic Factors Influencing Soil Carbon Stocks: A Global Assessment.” Mitigation and Adaptation Strategies for Global Change 25: 1129–1148. 10.1007/s11027-020-09926-1.

[gcb70135-bib-0033] Etter, A. , C. McAlpine , and H. Possingham . 2008. “Historical Patterns and Drivers of Landscape Change in Colombia Since 1500: A Regionalized Spatial Approach.” Annals of the Association of American Geographers 98, no. 1: 2–23. 10.1080/00045600701733911.

[gcb70135-bib-0034] Feldpausch, T. R. , L. Carvalho , K. D. Macario , et al. 2022. “Forest Fire History in Amazonia Inferred From Intensive Soil Charcoal Sampling and Radiocarbon Dating.” Forests and Global Change 5: 815438. 10.3389/ffgc.2022.815438.

[gcb70135-bib-0035] Fick, S. E. , and R. J. Hijmans . 2017. “WorldClim 2: New 1km Spatial Resolution Climate Surfaces for Global Land Areas.” International Journal of Climatology 37, no. 12: 4302–4315. 10.1002/joc.5086.

[gcb70135-bib-0036] FIRMS . 2015. Fire Information of Resource Management System Active Fire Data, Collection 4. Accessed January 15, 2015. https://earthdata.nasa.gov/data/near‐real‐time‐data/firms/active‐fire‐data.

[gcb70135-bib-0037] Gale, N. V. , and S. C. Thomas . 2021. “Spatial Heterogeneity in Soil Pyrogenic Carbon Mediates Tree Growth and Physiology Following Wildfire.” Journal of Ecology 109, no. 3: 1479–1490. 10.1111/1365-2745.13571.

[gcb70135-bib-0038] Gao, S. , C. Eisenberg , S. L. Morford , and T. H. DeLuca . 2024. “Fire Exclusion, Pyrogenic Carbon, and Ecosystem Function: What Have We Lost?” Anthropocene 46: 100438. 10.1016/j.ancene.2024.100438.

[gcb70135-bib-0039] Gee, G. W. , and J. W. Bauder . 1986. “Particle‐Size Analysis.” Methods of Soil Analysis: Part 1 Physical and Mineralogical Methods 5: 383–411. 10.2136/sssabookser5.1.2ed.c15.

[gcb70135-bib-0040] Hilscher, A. , and H. Knicker . 2011. “Degradation of Grass‐Derived Pyrogenic Organic Material, Transport of the Residues Within a Soil Column and Distribution in Soil Organic Matter Fractions During a 28‐Month Microcosm Experiment.” Organic Geochemistry 42, no. 1: 42–54. 10.1016/j.orggeochem.2010.10.005.

[gcb70135-bib-0041] Hobley, E. 2019. “Vertical Distribution of Soil Pyrogenic Matter: A Review.” Pedosphere 29, no. 2: 137–149. 10.1016/S1002-0160(19)60795-2.

[gcb70135-bib-0042] Hofstede, R. 1995. “The Effects of Grazing and Burning on Soil and Plant Nutrient Concentrations in Colombian páramo Grasslands.” Plant and Soil 173, no. 1: 111–132. 10.1007/BF00155524.

[gcb70135-bib-0043] Instituto Geografico Agustin Codazzi . 2001. Mapa Digital de Suelos del Departamento de Cundinamarca, República de Colombia. Escala 1:100.000. IGAC.

[gcb70135-bib-0044] Instituto Geografico Agustin Codazzi . 2002. Estudio General de Suelos del Departamento de Santander, República de Colombia. Escala 1:100.000. IGAC.

[gcb70135-bib-0045] Instituto Geografico Agustin Codazzi . 2005. Estudio general de suelos y zonificación de tierras del departamento de Boyacá. IGAC.

[gcb70135-bib-0046] IPCC . 2021. Climate Change 2021: The Physical Science Basis. Contribution of Working Group I to the Sixth Assessment Report of the Intergovernmental Panel on Climate Change. Cambridge University Press.

[gcb70135-bib-0047] Jackson, R. B. , K. Lajtha , S. E. Crow , G. Hugelius , M. G. Kramer , and G. Piñeiro . 2017. “The Ecology of Soil Carbon: Pools, Vulnerabilities, and Biotic and Abiotic Controls.” Annual Review of Ecology, Evolution, and Systematics 48, no. 1: 419–445. 10.1146/annurev-ecolsys-112414054234.

[gcb70135-bib-0048] Jantalia, C. P. , D. V. Resck , B. J. Alves , L. Zotarelli , S. Urquiaga , and R. M. Boddey . 2007. “Tillage Effect on C Stocks of a Clayey Oxisol Under a Soybean‐Based Crop Rotation in the Brazilian Cerrado Region.” Soil and Tillage Research 95, no. 1–2: 97–109. 10.1016/j.still.2006.11.005.

[gcb70135-bib-0049] Jantz, N. , and H. Behling . 2012. “A Holocene Environmental Record Reflecting Vegetation, Climate, and Fire Variability at the Páramo of Quimsacocha, Southwestern Ecuadorian Andes.” Vegetation History and Archaeobotany 21: 169–185. 10.1007/s00334-011-0327-x.

[gcb70135-bib-0050] Jauss, V. , M. Johnson , E. Krull , M. Daub , and J. Lehmann . 2015. “Pyrogenic Carbon Controls Across a Soil Catena in the Pacific Northwest.” Catena 12: 53–59. 10.1016/j.catena.2014.09.001.

[gcb70135-bib-0051] Jobbágy, E. G. , and R. B. Jackson . 2000. “The Vertical Distribution of Soil Organic Carbon and Its Relation to Climate and Vegetation.” Ecological Applications 10, no. 2: 423–436. 10.1890/1051-0761(2000)010[0423:TVDOSO]2.0.CO;2.

[gcb70135-bib-0052] Johnson, D. , J. D. Murphy , R. F. Walker , D. W. Glass , and W. W. Miller . 2007. “Wildfire Effects on Forest Carbon and Nutrient Budgets.” Ecological Engineering 31, no. 3: 183–192. 10.1016/j.ecoleng.2007.03.003.

[gcb70135-bib-0053] Jones, M. W. , C. Santín , G. R. de van Werf , and S. H. Doerr . 2019. “Global Fire Emissions Buffered by the Production of Pyrogenic Carbon.” Nature Geoscience 12, no. 9: 742–747. 10.1038/s41561-019-0403-x.

[gcb70135-bib-0054] Kim, K. H. , J. Y. Kim , T. S. Cho , and J. W. Choi . 2012. “Influence of Pyrolysis Temperature on Physicochemical Properties of Biochar Obtained From the Fast Pyrolysis of Pitch Pine ( *Pinus rigida* ).” Bioresource Technology 118: 158–162. 10.1016/j.biortech.2012.04.094. 22705519

[gcb70135-bib-0055] Klein, D. , J. P. Fuentes , A. Schmidt , H. Schmidt , and A. Schulte . 2008. “Soil Organic C as Affected by Silvicultural and Exploitative Interventions in Nothofagus Pumilio Forests of the Chilean Patagonia.” Forest Ecology and Management 255, no. 10: 3549–3555. 10.1016/j.foreco.2008.03.002.

[gcb70135-bib-0056] Koch, A. , C. Brierley , M. M. Maslin , and S. L. Lewis . 2019. “Earth System Impacts of the European Arrival and Great Dying in the Americas After 1492.” Quaternary Science Reviews 207: 13–36. 10.1016/j.quascirev.2018.12.004.

[gcb70135-bib-0057] Koele, N. , M. Bird , J. Haig , et al. 2017. “Amazon Basin Forest Pyrogenic Carbon Stocks: First Estimate of Deep Storage.” Geoderma 306: 237–243. 10.1016/j.geoderma.2017.07.029.

[gcb70135-bib-0058] Lehmann, J. , J. Skjemstad , S. Sohi , et al. 2008. “Australian Climate–Carbon Cycle Feedback Reduced by Soil Black Carbon.” Nature Geoscience 1, no. 12: 832–835.

[gcb70135-bib-0111] Malone, B. P. , A. B. McBratney , B. Minasny , and G. M. Laslett . 2009. “Mapping Continuous Depth Functions of Soil Carbon Storage and Available Water Capacity.” Geoderma 154, no. 1‐2: 138–152. 10.1016/j.geoderma.2009.10.007.

[gcb70135-bib-0059] Marcelino, V. , C. E. Schaefer , and G. Stoops . 2018. “Oxic and Related Materials.” In Interpretation of Micromorphological Features of Soils and Regoliths, edited by G. Stoops , 663–689. Elsevier. 10.1016/B978-0-444-63522-8.00023-1.

[gcb70135-bib-0060] Masiello, C. A. , and A. A. Berhe . 2020. “First Interactions With the Hydrologic Cycle Determine Pyrogenic Carbon's Fate in the Earth System.” Earth Surface Processes and Landforms 45, no. 10: 2394–2398. 10.1002/esp.4925.

[gcb70135-bib-0061] Matosziuk, L. M. , A. Gallo , J. Hatten , et al. 2020. “Short‐Term Effects of Recent Fire on the Production and Translocation of Pyrogenic Carbon in Great Smoky Mountains National Park.” Frontiers in Forests and Global Change 3: 6. 10.3389/ffgc.2020.00006.

[gcb70135-bib-0062] Mayer, M. , C. E. Prescott , W. E. Abaker , et al. 2020. “Tamm Review: Influence of Forest Management Activities on Soil Organic Carbon Stocks: A Knowledge Synthesis.” Forest Ecology and Management 466: 118127. 10.1016/j.foreco.2020.118127.

[gcb70135-bib-0063] McDowell, N. , C. D. Allen , K. Anderson‐Teixeira , et al. 2018. “Drivers and Mechanisms of Tree Mortality in Moist Tropical Forests.” New Phytologist 219, no. 3: 851–869. 10.1111/nph.15027. 29451313

[gcb70135-bib-0064] Meredith, W. , P. L. Ascough , M. I. Bird , et al. 2012. “Assessment of Hydropyrolysis as a Method for the Quantification of Black Carbon Using Standard Reference Materials.” Geochimica et Cosmochimica Acta 97: 131–147. 10.1016/j.gca.2012.08.037.

[gcb70135-bib-0065] Molano, J. 1996. “Environmental Problems of the Andean páramo.” In The páramo. Ecosystem to Protect. Serie montañas tropandinas Vol. II. Fundación Ecosistemas Andinos.

[gcb70135-bib-0066] Montes‐Pulido, C. , M. I. Bird , J. Serrano , C. Quesada , and T. R. Feldpausch . 2023. Dataset: Climatic and Edaphic Drivers of Soil Organic Carbon and Pyrogenic Carbon Stocks Across Elevation and Disturbance Gradients in Colombian Andean forests. 10.5281/zenodo.14926032. PMC12290775 40709364

[gcb70135-bib-0068] Montes‐Pulido, C. , T. R. Feldpausch , T. Pennington , M. Bird , and C. Quesada . 2023. Soil Nutrients, Bulk Density and Pyrogenic Carbon in Permanent Monitoring Plots Along Altitudinal and Forest Perturbation Gradients in the Colombian Andes, 2019–2022. NERC EDS Environmental Information Data Centre. 10.5285/fbcfc877-a38e-4c26-8d8b-7226392493db.

[gcb70135-bib-0069] Nepstad, D. , G. Carvalho , A. C. Barros , et al. 2001. “Road Paving, Fire Regime Feedbacks, and the Future of Amazon Forests.” Forest Ecology and Management 154, no. 3: 395–407. 10.1016/S0378-1127(01)00511-4.

[gcb70135-bib-0070] Nguyen, B. T. , and J. Lehmann . 2009. “Black Carbon Decomposition Under Varying Water Regimes.” Organic Geochemistry 40, no. 8: 846–853. 10.1016/j.orggeochem.2009.05.004.

[gcb70135-bib-0071] Nottingham, A. T. , P. Meir , E. Velasquez , and B. L. Turner . 2020. “Soil Carbon Loss by Experimental Warming in a Tropical Forest.” Nature 584, no. 7820: 234–237. 10.1038/s41586-020-2566-4. 32788738

[gcb70135-bib-0072] Oliveira, E. A. , B. H. Marimon‐Junior , B. S. Marimon , et al. 2020. “Legacy of Amazonian Dark Earth Soils on Forest Structure and Species Composition.” Global Ecology and Biogeography 29, no. 9: 1458–1473. 10.1111/geb.13116.

[gcb70135-bib-0073] Patiño, S. , Y. Hernández , C. Plata , et al. 2021. “Influence of Land Use on Hydro‐Physical Soil Properties of Andean páramos and Its Effect on Streamflow Buffering.” Catena 202: 105227. 10.1016/j.catena.2021.105227.

[gcb70135-bib-0074] Patiño, V. M. 1965. History of Farming in Equinoctial America. Imprenta Departamental.

[gcb70135-bib-0075] Pérez‐Escobar, O. A. , A. Zizka , M. A. Bermúdez , et al. 2022. “The Andes Through Time: Evolution and Distribution of Andean Floras.” Trends in Plant Science 27, no. 4: 364–378. 10.1016/j.tplants.2021.09.010. 35000859

[gcb70135-bib-0076] Peter‐Contesse, H. , K. Lajtha , A. Boettcher , R. O'Kelley , and A. Mayedo . 2024. “Unearthing the Legacy of Wildfires: Post Fire Pyrogenic Carbon and Soil Carbon Persistence Across Complex Pacific Northwest Watersheds.” Biogeochemistry: 1–18. 10.1007/s10533-024-01151-1.

[gcb70135-bib-0110] Pleysier, J. L. , and A. S. R. Juo . 1980. “A Single‐Extraction Method Using Silver‐Thiourea for Measuring Exchangeable Cations and Effective CEC in Soils with Variable Charges.” Soil Science 129, no. 4: 205–211. 10.1097/00010694-198004000-00002.

[gcb70135-bib-0077] Ponomarenko, E. V. , and D. W. Anderson . 2001. “Importance of Charred Organic Matter in Black Chernozem Soils of Saskatchewan.” Canadian Journal of Soil Science 81, no. 3: 285–297. 10.4141/S00-075.

[gcb70135-bib-0078] Prates, L. , G. G. Politis , and S. I. Perez . 2020. “Rapid Radiation of Humans in South America After the Last Glacial Maximum: A Radiocarbon‐Based Study.” PLoS One 15, no. 7: e0236023. 10.1371/journal.pone.0236023. 32697794 PMC7375534

[gcb70135-bib-0079] Pressler, Y. , C. M. Boot , S. Abiven , E. Lugato , M. F. Cotrufo , and M. Farrell . 2022. “Continental‐Scale Measurements of Soil Pyrogenic Carbon in Europe.” Soil Research 60, no. 2: 103–113. 10.1071/SR19396.

[gcb70135-bib-0080] Qi, F. , R. Naidu , N. S. Bolan , et al. 2017. “Pyrogenic Carbon in Australian Soils.” Science of the Total Environment 586: 849–857. 10.1016/j.scitotenv.2017.02.064. 28215804

[gcb70135-bib-0081] Quesada, C. A. , J. Lloyd , M. Schwarz , et al. 2010. “Variations in Chemical and Physical Properties of Amazon Forest Soils in Relation to Their Genesis.” Biogeosciences 7, no. 5: 1515–1541. 10.5194/bg-7-1515-2010.

[gcb70135-bib-0082] R Core Team . 2022. R: A Language and Environment for Statistical Computing. R Foundation for Statistical Computing. https://www.r‐project.org/.

[gcb70135-bib-0083] Raich, J. W. , A. E. Russell , K. Kitayama , W. J. Parton , and P. M. Vitousek . 2006. “Temperature Influences Carbon Accumulation in Moist Tropical Forests.” Ecology 87, no. 1: 76–87. 10.1890/05-0023. 16634298

[gcb70135-bib-0084] Rappaport, D. I. , D. C. Morton , M. Longo , M. Keller , R. Dubayah , and M. N. dos‐Santos . 2018. “Quantifying Long‐Term Changes in Carbon Stocks and Forest Structure From Amazon Forest Degradation.” Environmental Research Letters 13, no. 6: 065013. 10.1088/1748-9326/aac331.

[gcb70135-bib-0085] Reisser, M. , R. S. Purves , M. W. Schmidt , and S. Abiven . 2016. “Pyrogenic Carbon in Soils: A Literature‐Based Inventory and a Global Estimation of Its Content in Soil Organic Carbon and Stocks.” Frontiers in Earth Science 4: 80. 10.3389/feart.2016.00080.

[gcb70135-bib-0086] Rivas, Y. , F. Matus , C. Rumpel , H. Knicker , and E. Garrido . 2012. “Black Carbon Contribution in Volcanic Soils Affected by Wildfire or Stubble Burning.” Organic Geochemistry 47: 41–50. 10.1016/j.orggeochem.2012.03.007.

[gcb70135-bib-0087] Robichaud, P. R. 2000. “Fire and Erosion: Evaluating the Effectiveness of a Post‐Fire Rehabilitation Treatment, Contour‐Felled Logs.” In Watershed Management and Operations Management 2000, 1–11. 10.1061/40499(2000)36.

[gcb70135-bib-0088] Rodríguez Eraso, N. , D. Armenteras‐Pascual , and J. R. Alumbreros . 2013. “Land Use and Land Cover Change in the Colombian Andes: Dynamics and Future Scenarios.” Journal of Land Use Science 8, no. 2: 154–174. 10.1080/1747423X.2011.650228.

[gcb70135-bib-0089] Rolando, J. L. , J. C. Dubeux Jr. , W. Perez , et al. 2017. “Soil Organic Carbon Stocks and Fractionation Under Different Land Uses in the Peruvian High‐Andean Puna.” Geoderma 307: 65–72. 10.1016/j.geoderma.2017.07.037.

[gcb70135-bib-0090] Román‐Cuesta, R. M. , C. Carmona‐Moreno , G. Lizcano , et al. 2014. “Synchronous Fire Activity in the Tropical High Andes: An Indication of Regional Climate Forcing.” Global Change Biology 20, no. 6: 1929–1942. 10.1111/gcb.12538. 24464954

[gcb70135-bib-0091] Rumpel, C. , J. Leifeld , C. Santín , and S. H. Doerr . 2015. “Movement of Biochar in the Environment.” In Biochar for Environmental Management. Science, Technology and Implementation, edited by J. L. Lehmann and S. Joseph , 2nd ed., 283–299. Routledge, Taylor & Francis.

[gcb70135-bib-0092] Saiz, G. , I. Goodrick , C. Wurster , P. N. Nelson , J. Wynn , and M. Bird . 2018. “Preferential Production and Transport of Grass‐Derived Pyrogenic Carbon in NE‐Australian Savanna Ecosystems.” Frontiers in Earth Science 5: 115. 10.3389/feart.2017.00115.

[gcb70135-bib-0093] Santín, C. , S. H. Doerr , E. S. Kane , et al. 2016. “Towards a Global Assessment of Pyrogenic Carbon From Vegetation Fires.” Global Change Biology 22, no. 1: 76–91. 10.1111/gcb.12985. 26010729

[gcb70135-bib-0094] Schawe, M. , S. Glatzel , and G. Gerold . 2007. “Soil Development Along an Altitudinal Transect in a Bolivian Tropical Montane Rainforest: Podzolization vs. Hydromorphy.” Catena 69, no. 2: 83–90. 10.1016/j.catena.2006.04.023.

[gcb70135-bib-0095] Schiferl, J. D. , M. B. Bush , M. R. Silman , and D. H. Urrego . 2018. “Vegetation Responses to Late Holocene Climate Changes in an Andean Forest.” Quaternary Research 89, no. 1: 60–74. 10.1017/qua.2017.64.

[gcb70135-bib-0096] Schmidt, M. W. I. , M. S. Torn , S. Abiven , et al. 2011. “Persistence of Soil Organic Matter as an Ecosystem Property.” Nature 478: 49–56. 10.1038/nature10386. 21979045

[gcb70135-bib-0097] Tiessen, H. , and J. O. Moir . 1993. “Total and Organic Carbon.” In: Soil Sampling and Methods of Analysis, edited by M. E. Carter , 187–199. Lewis.

[gcb70135-bib-0098] Turcios, M. M. , M. M. Jaramillo , J. F. do Vale Jr. , P. M. Fearnside , and R. I. Barbosa . 2016. “Soil Charcoal as Long‐Term Pyrogenic Carbon Storage in Amazonian Seasonal Forests.” Global Change Biology 22, no. 1: 190–197. 10.1111/gcb.13049. 26207816

[gcb70135-bib-0099] U.S. Geological Survey . n.d. EarthExplorer. Accessed November 13, 2021. https://earthexplorer.usgs.gov/.

[gcb70135-bib-0100] Van der Hammen, T. , J. H. Werner , and H. van Dommelen . 1973. “Palynological Record of the Upheaval of the Northern Andes: A Study of the Pliocene and Lower Quaternary of the Colombian Eastern Cordillera and the Early Evolution of Its High‐Andean Biota.” Review of Palaeobotany and Palynology 16: 1–122.

[gcb70135-bib-0101] Villabona, D. A. , and D. A. Pascual . 2012. “Incidencia de incendios sobre la vegetación de Cundinamarca y Bogotá DC (Colombia), entre 2001 y 2010.” Acta Biológica Colombiana 17, no. 1: 143–157.

[gcb70135-bib-0102] Vogel, M. M. , R. Orth , F. Cheruy , et al. 2017. “Regional Amplification of Projected Changes in Extreme Temperatures Strongly Controlled by Soil Moisture‐Temperature Feedbacks.” Geophysical Research Letters 44, no. 3: 1511–1519. 10.1002/2016GL071235.

[gcb70135-bib-0103] Volkova, L. , H. Krisnawati , W. C. Adinugroho , et al. 2021. “Identifying and Addressing Knowledge Gaps for Improving Greenhouse Gas Emissions Estimates From Tropical Peat Forest Fires.” Science of the Total Environment 763: 142933. 10.1016/j.scitotenv.2020.142933. 33268261

[gcb70135-bib-0105] Yang, S. , E. Cammeraat , B. Jansen , M. den Haan , E. van Loon , and J. Recharte . 2018. “Soil Organic Carbon Stocks Controlled by Lithology and Soil Depth in a Peruvian Alpine Grassland of the Andes.” Catena 171: 11–21. 10.1016/j.catena.2018.06.038.

[gcb70135-bib-0106] Zaffar, M. , and L. U. Sheng‐Gao . 2015. “Pore Size Distribution of Clayey Soils and Its Correlation With Soil Organic Matter.” Pedosphere 25, no. 2: 240–249. 10.1016/S1002-0160(15)60009-1.

[gcb70135-bib-0107] Zhan, C. , J. Cao , Y. Han , et al. 2013. “Spatial Distributions and Sequestrations of Organic Carbon and Black Carbon in Soils From the Chinese Loess Plateau.” Science of the Total Environment 465: 255–266. 10.1016/j.scitotenv.2012.10.113. 23219202

[gcb70135-bib-0108] Zhao, Y. F. , X. Wang , S. L. Jiang , et al. 2021. “Climate and Geochemistry Interactions at Different Altitudes Influence Soil Organic Carbon Turnover Times in Alpine Grasslands.” Agriculture, Ecosystems & Environment 320: 107591. 10.1016/j.agee.2021.107591.

[gcb70135-bib-0109] Zimmermann, M. , M. I. Bird , C. Wurster , et al. 2012. “Rapid Degradation of Pyrogenic Carbon.” Global Change Biology 18, no. 11: 3306–3316. 10.1111/j.1365-2486.2012.02796.x.

