# Supplementary material for: Climatic and Edaphic Drivers of Soil Organic Carbon and Pyrogenic Carbon Stocks Across Elevation and Disturbance Gradients in Colombian Andean Forests
Source: Glob Chang Biol. 2025 Jul 25;31(7):e70135. doi: 10.1111/gcb.70135 (PMC12290775; doi:10.1111/gcb.70135)

**Supplementary material**

**Climatic and edaphic drivers of soil organic carbon and pyrogenic carbon stocks across elevation and disturbance gradients in Colombian Andean forests**

Carmen Montes-Pulido^1^, Michael I. Bird^2^, Lidiany C. da Silva Carvalho^5^ Julieth Serrano ^3,5^, Carlos Quesada^4^, Ted R. Feldpausch ^5^

^1^ School of Agricultural, Livestock, and Environmental Sciences, Universidad Nacional Abierta y a Distancia (UNAD), Bogota, Colombia

^2^ James Cook University, Cairns, Australia

^3^ Fauna & Flora International, Cambridge, UK

^4^ Instituto Nacional de Pesquisas da Amazônia (INPA), Manaus, Brazil

^5^ Geography, Faculty of Environment, Science and Economy, University of Exeter, Exeter, UK


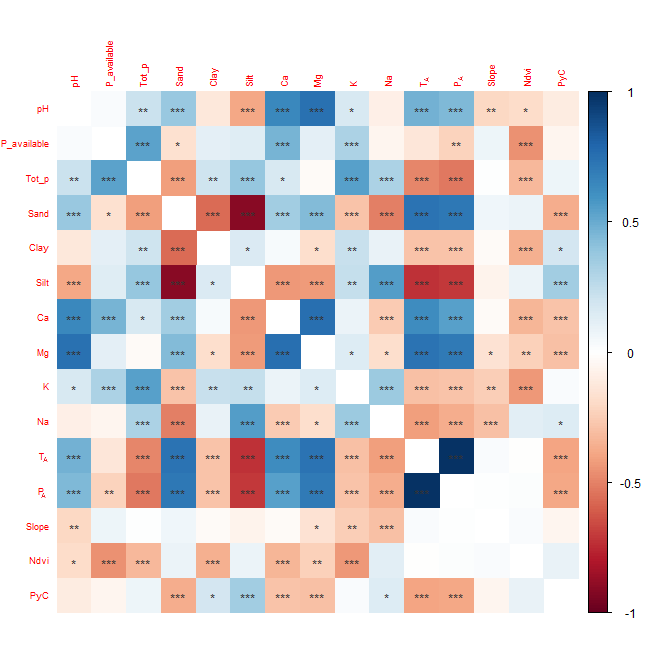
**Figure S1**. Correlation between PyC, NDVI, slope, climatic (Annual mean temperature-AMT-, Annual precipitation -AP-) and soil variables (pH, P-available, total_ P Sand, Silt, Ca, Mg, K and Na)

**Kruskal-Wallis test results:**

PyC x soil Depth:

Kruskal-Wallis chi-squared = 8.166, df = 4, p-value = 0.09

PyC x Disturbance:

Kruskal-Wallis chi-squared = 1.1218, df = 3, p-value = 0.77

PyC x elevation zones:

Kruskal-Wallis chi-squared = 111.8588, df = 2, p-value = 0.00001

**Table S1**. Comparisons between elevation zones

| Comparisons elevation zones | t | p |
| --- | --- | --- |
| Low Andes x High Andes | 10.47 | < 0.0001 |
| Low Andes x Medium Andes | -3.89 | 0.0001 |
| High Andes x Medium Andes | 6.44 | <0.0001 |

Significant differences in PyC values were observed across elevation zones

No significant differences in PyC values were observed across depths and disturbance

**Table S2**. Generalised linear model fitting

| Predictors | AIC | R2 |
| --- | --- | --- |
| PA + pH + Ca + NDVI + Clay | 34.98 | 0.56 |
| PA + NDVI + Clay | 35.28 | 0.50 |
| PA + pH + Ca + Clay | 35.37 | 0.52 |
| PA + pH + NDVI + Clay | 35.40 | 0.52 |
| PA + K + NDVI + Clay | 35.67 | 0.52 |

Estimated model equation:

PyC ~ PA + pH + Ca + NDVI + clay

**Figure S2**: Main edaphic and climatic drivers of pyrogenic carbon (PyC) across elevation gradients in Colombian Andean forest. Panel (a) Represents relationship between PyC and Clay (%); (b) represents relationship between PyC (%) and mean anual precipitation (mm year ^-1^). Data from 36 forest and agrosilvopastoral plots of 0.5 ha at three elevation zones.


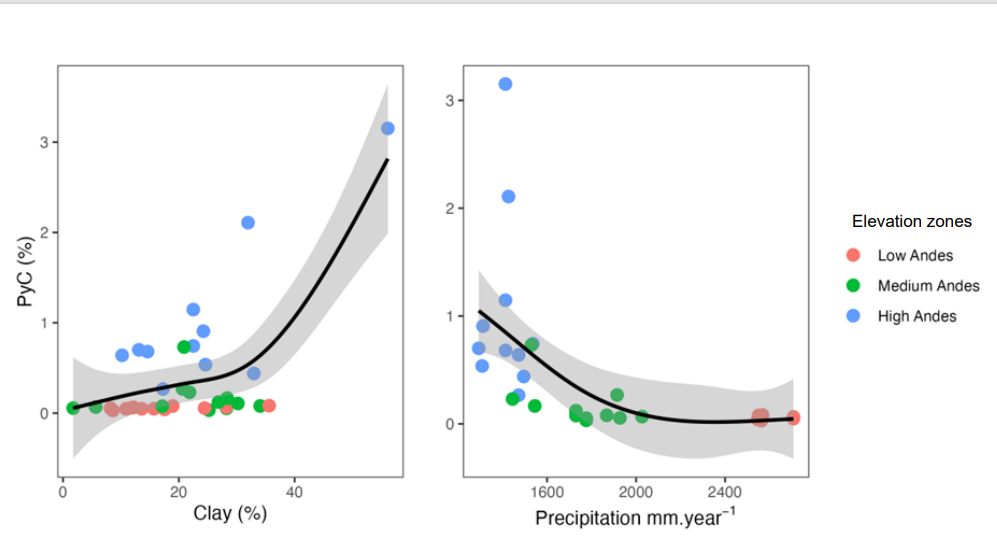

Supplement: Supplementary file 1 — Data S1. [file GCB-31-e70135-s001.docx]
